# Augmented reality-driven organ deformation modeling: a roadmap for next-generation intraoperative navigation

**DOI:** 10.1097/MS9.0000000000004243

**Published:** 2025-11-04

**Authors:** Muhammad Umer Suleman, Muhammad Mursaleen, Umer Khalil, Syeda Nafisa Tabassum

**Affiliations:** a Department of Internal Medicine, Ayub Medical College, Abbottabad, Pakistan; b Department of Microbiology, Bangladesh Rural Advancement Committee, Dhaka, Bangladesh

**Keywords:** augmented reality, clinical validation, image registration, intraoperative navigation, organ deformation modeling, soft tissue, surgical training

## Abstract

Accurate intraoperative modeling of organ deformation is essential for translating augmented reality (AR) guidance into measurable clinical benefit. This editorial evaluates deformation-aware AR systems for intraoperative guidance, emphasizing how precise deformation modeling can reduce targeting errors, shorten operative time, and lower procedure-related complications. We review three principal algorithmic strategies: physics-based biomechanical models (e.g., finite-element methods), data-driven image-registration and deep-learning techniques, and hybrid sensor-informed frameworks and summarize representative applications in neurosurgery (brain-shift compensation), hepatic, and thoracic interventions. Key translational barriers are discussed: real-time performance, validation against objective error metrics, workflow integration, regulatory pathways, and equitable access. We also address ethical considerations and data stewardship. We recommend prospective clinical trials with measurable endpoints (resection-margin accuracy, intraoperative blood loss, conversion rates, operative duration, and length of stay) and propose a translational roadmap emphasizing multimodal integration, standardization, and clinician training. Coordinated technical and clinical validation could establish deformation-aware AR as a validated intraoperative tool to improve surgical precision and patient outcomes.

## Introduction

Augmented reality (AR) offers surgeons an unprecedented ability to superimpose virtual anatomical models onto the operative field, enhancing spatial understanding and precision^[1,2]^. In practice, AR systems overlay patient-specific 3D reconstructions [from computed tomography (CT)/magnetic resonance imaging] onto endoscopic or open views, with the promise of “x-ray vision” through tissues^[3,4]^. However, unlike rigid anatomy (e.g., bone), soft tissues deform significantly during surgery due to respiration, traction, pneumoperitoneum, or tumor resection. Static preoperative models thus misalign with the intraoperative reality unless deformation is explicitly addressed^[1,5]^. Despite AR’s potential to improve visualization and targeting, clinical utility critically depends on maintaining alignment between virtual overlays and the patient’s dynamically changing anatomy, complicated by soft-tissue deformation from respiration, traction, resection, and insufflation. Organ deformation modeling (ODM) refers to computational methods that predict or measure intraoperative soft-tissue shape changes and update virtual anatomy in real time so AR overlays remain registered to the surgical scene^[1]^. ODM algorithms aim to predict and update soft-tissue shape in real time, enabling next-generation AR navigation with maintained accuracy. This editorial reviews the current global state of AR-guided navigation with deformation modeling, delineates major algorithmic approaches, and outlines the translational roadmap, spanning technical, clinical, regulatory, and ethical facets, toward routine deployment. In preparation of this article, AI-assisted tools were used in accordance with the TITAN Guidelines 2025^[6]^.

## Technical approaches to ODM

Organ deformation can be modeled with broadly three algorithmic strategies: model-based (biomechanical), data-driven (learning or image-based), and hybrid methods. Model-based approaches use physics or geometry to simulate tissue response, typically finite-element (FEM), a physics-based numerical technique that discretizes tissue into small elements to simulate stress/strain or mass-spring models. For example, Golse *et al* developed a real-time FEM liver model driven by RGB-D camera data during open hepatectomy^[3]^. In their system, an elastic liver mesh deforms according to external forces sensed by a depth camera, producing AR overlays of vascular anatomy; clinical feasibility was demonstrated with a ~7.9 mm root-mean-square error against internal fiducials^[3]^. FEM models often achieve high accuracy for realistic tissue elasticity but can be computationally intensive. Simplified physics-based models (e.g., linear elasticity) or statistical shape models can reduce complexity but may sacrifice precision or require extensive precomputation.

Data-driven techniques avoid explicit physics by inferring deformations from image data or learned models. Classical 3D–2D registration methods align preoperative 3D models to intraoperative images via landmark or intensity matching. For instance, in Medical Image Computing and Computer-Assisted Intervention (MICCAI) conference 2022, a challenge “Preoperative-to-Intraoperative Laparoscopic Fusion” (P2ILF) showed that current AR liver navigation relies on manually annotated anatomical landmarks (e.g., falciform ligament, hepatic contours) to register CT-based liver models to laparoscopic video^[7]^. Deep learning (DL) has begun to automate this process: teams in P2ILF used convolutional networks to segment 2D laparoscopic landmarks and differentiable renderers (renderers that permit gradients to be computed from image-space losses back to model or pose parameters, enabling end-to-end learning) for registration^[7]^. More generally, DL-based deformable registration networks, e.g., VoxelMorph (a DL architecture that predicts dense volumetric displacement fields between image volumes, enabling fast deformable registration) variants can predict dense displacement fields between image volumes, potentially handling complex deformations from respiration or surgical manipulation. Image registration methods (both iterative and learning-based) can run in sub-second times with Graphics Processing Unit (GPU) acceleration, but often struggle with large, nonuniquely identifiable shifts in feature-poor organs.

Hybrid methods combine both paradigms: for example, one can use sparse landmarks or ultrasound (US) updates to drive a biomechanical model. Pelanis *et al* fused intraoperative fluoroscopy with tracked laparoscopic instruments to register a deformable liver model^[8]^. Smit *et al* combined electromagnetic-tracked US with a liver sensor to compensate for respiratory motion, yielding target registration errors ≈8–10 mm^[9]^. By coupling FEM or statistical models with real-time sensor data (US, fiducials, optical tracking), hybrids exploit the physical plausibility of models while correcting via data. Table 1 summarizes representative methods by category, illustrative use cases, and relative trade-offs.

**Table 1:** Representative methods categorized by type, with illustrative use cases and a summary of their relative trade-offs

Method category	Representative techniques	Example use case	Pros	Cons
Model-based	Finite-element models (FEM) of tissue elasticity^[3]^; mass-spring meshes; physics-based simulation^[3]^	Brain shift compensation in neurosurgery^[10]^; open liver resection with depth-sensor updates^[3]^	High-fidelity physical modeling of tissue properties; explainable behavior	Computationally expensive; requires parameter estimation (e.g., material properties
Data-driven (image-based)	3D–2D image registration (landmark, surface matching)^[4,7]^; optical/sensor tracking; deep neural nets for deformable registration^[4,7]^	Video-assisted thoracoscopic surgery lung segmentectomy: auto-registration of 3D pulmonary model onto stereo endoscope^[4]^; laparoscopic tumor overlay via fiducials^[8]^	Fast inference (especially with DL); adaptable to imaging modalities (CT, US, endoscope)	May be less robust for large or unseen deformations; requires good image features or training data
Hybrid	Landmark-driven FEM (e.g., FEM updated by tracked points)^[3,9]^; ML-assisted physics (e.g., neural nets initializing a model)	Laparoscopic liver navigation: intra-op CT/fluoroscopy updates liver model^[8]^; ultrasound-based liver tracking^[9]^	Balances accuracy and speed; data corrects model drift	More complex to implement; requires integrated hardware (tracking, US, etc.)

Representative ODM approaches for AR navigation. Model-based methods (top) yield high accuracy but need computational resources; data-driven (middle) rely on images or machine learning (ML) for speed; hybrids (bottom) integrate both. Examples drawn from the literature^[3,4,8]^.

## Clinical applications of AR with deformation compensation

Deployment status varies by specialty; many systems remain in prototype or single-center pilot stages, a few have reached early clinical feasibility testing, and only a small subset has been evaluated in multicenter clinical studies.

### Neurosurgery

Brain tumor resection and cerebrovascular surgery are prime AR targets due to critical 3D anatomy and the problem of *brain shift*. Intraoperative US or tracked pointers can update a biomechanical brain model. For example, combining AR with an US-derived deformation model has been shown to improve visualization accuracy in resecting gliomas^[10]^. AR neuronavigation (e.g., via microscope or Head-Up Display has been used in endoscopic third ventriculostomy and transsphenoidal pituitary surgery, improving accuracy^[2]^. In one series, AR guidance reduced target registration error from ~1.02 mm (standard) to ~0.76 mm (AR-assisted) in pituitary cases^[2]^. Such gains, though small in absolute terms, can translate into safer resections of critical structures. However, routine use is limited by workflow integration: stereoscopic cameras, patient-mounted trackers, and real-time modeling must be added to delicate cranial operations without interrupting care.

### Liver surgery

The deformable liver (and other abdominal organs) has motivated many AR navigation studies. Laparoscopic liver resection is especially challenging because pneumoperitoneum and instrument retraction deform the liver surface. Pelanis *et al* demonstrated an AR navigation platform where intra-op fluoroscopy and optical tracking continuously registered the liver model, achieving ~3.8 mm surface fiducial error around lesions^[8]^. Teatini *et al* showed that AR systems that incorporate intraoperative imaging (CT/fluoroscopy) significantly outperform pre-op-only guidance: without updating, insufflation shifts cause marked navigation errors^[5]^. In bench and animal studies, AR overlay of 3D liver vasculature improved tumor localization accuracy markedly, reducing targeting errors from tens of millimeters to under 10 mm^[11]^. Similar approaches have been applied in open hepatectomy: Golse *et al* used a depth sensor to drive an FEM liver model, reporting *real-time* overlays of anatomy with sub-centimeter accuracy (mean Root Mean Square ~7.9 mm)^[3]^. US-guided methods show promise, too: Smit *et al* used tracked US and an electromagnetic liver-mounted sensor to compensate motion, achieving mean target errors ~8.5 mm^[9]^. In all these cases, AR interfaces [monitors or head-mounted displays (HMDs)] allowed the surgeon to “see” the tumor and vessels despite organ motion.

### Lung and thoracic surgery

AR has been explored in minimally invasive lung resections, where the collapsed lung deforms unpredictably. Peek *et al* (2025) presented an AR workflow for video/robotic thoracoscopy: stereoscopic endoscopic imagery was used to generate a live 3D point cloud of the deflated lung, onto which a CT-derived segmental model was registered. This yielded extremely precise overlays (median surface error ~0.20 mm) and allowed visualization of invisible bronchovascular targets in eight patients^[4]^. Such work highlights that even rigidish structures (bronchi, vessels) can be localized via AR if smart registration compensates for deflation and retraction. Future AR tools in thoracic surgery may combine such vision-based registration with precomputed FEM lung models to anticipate deformation during ventilation.

### Other domains

AR is also being tested in abdominal interventions (kidney tumor ablation, pancreas surgery), cardiac (congenital heart defect overlays on beating heart), and spine (e.g., pedicle screw placement, though spine is mostly rigid). We note: US Food and Drug Administration (FDA)-cleared AR devices already exist in orthopedics (AR knee and spinal guides) and ENT, paving regulatory paths^[12]^. However, soft-tissue AR (requiring deformation handling) lags behind, largely confined to research settings.

Across specialties, imaging modality affects approach: Endoscopic video and surface scanning excel for visible deformations; intraoperative CT/fluoroscopy or US are invaluable for internal alignment. HMDs (like HoloLens) and optical see-through displays ease surgeon interaction. In many studies, AR systems are evaluated on phantoms or small patient series, showing improved targeting accuracy (often assessed by target registration error) and subjective ease of use^[2,11]^. Yet, large clinical trials of AR-based deformation models are rare.

## Limitations and translational challenges

Despite the technical promise, several hurdles impede widespread AR deformation navigation:

## Computational constraints

Physics-based simulation (e.g., full FEM) produces physically plausible deformation but is computationally demanding; conversely, many data-driven registration methods are fast but can produce implausible outputs when faced with out-of-distribution anatomy or unmodeled forces. In liver laparoscopy, even rigid landmark alignment has shown errors >8 mm without deformation compensation, demonstrating the sensitivity of guidance to unmodeled motion. Mitigations include model-order reduction, regional updates (update only the surgical target region), GPU acceleration, and hybrid schemes that combine fast learned priors with local physics corrections. Systems should report latency, update frequency, and uncertainty, and provide clear visual cues and simple manual overrides when fidelity degrades^[5]^.

## Clinical and workflow integration

Adding tracking cameras, fiducials, or extra intraoperative imaging increases OR complexity, sterility burden, and cognitive load; HMD drift, specular surfaces, and mode-switching (endoscopic → open) are practical failure modes. Addressing these issues requires iterative human-factors design, minimal and context-sensitive overlays, simulated-OR testing, and straightforward manual override policies. Pilot studies must measure human-centered outcomes (surgeon workload, task time, override frequency) in addition to technical metrics such as TRE or surface-RMS^[12]^.

## Regulatory, validation, and standardization

ODM-enabled AR systems are typically regulated as SaMD or integrated device–software systems and demand staged validation (bench/phantom → feasibility → prospective clinical studies). The field currently lacks a single accepted benchmark for AR accuracy; studies use varied metrics (Target Registration Error, overlay error, clinical outcomes), complicating comparisons. Early regulatory engagement and participation in community benchmarking (e.g., MICCAI challenges) will help align on acceptable surrogate metrics and study designs^[7,12]^

## Equity, privacy, and commercialization

Wireless HMDs and cloud-based processing raise cybersecurity and privacy concerns; high-cost systems risk widening disparities if limited to tertiary centers. Mitigations include embedding cybersecurity/privacy-by-design, pursuing modular solutions that can run on commodity hardware where feasible, and including cost-effectiveness endpoints in validation programs^[12]^.

## Interdisciplinary collaboration: how engineers and surgeons must work differently

Siloed development engineers optimize algorithm metrics on curated datasets while clinicians focus on workflow, which undermines translation. Best practice is surgeon-led problem definition, engineer-led prototyping, and rapid simulated-OR cycles with both teams present. Co-define acceptance thresholds (technical and human-centered), embed clinician champions on development teams, and co-create annotated multimodal datasets with explicit governance to improve generalizability.

## Counterarguments and practical responses

A common critique is that deformation-aware AR will overcomplicate the OR. A pragmatic response is progressive integration: prioritize high-impact, low-risk indications where deformation materially alters decision-making; demonstrate objective surrogate improvements (e.g., TRE reductions) and human-factors safety in staged studies; then scale. For cost/equity concerns, favor modular, interoperable designs and publish cost-effectiveness alongside clinical results.

## Roadmap and future directions

To translate AR deformation modeling from the lab to OR, coordinated progress is needed on multiple fronts.

### Algorithmic advances

Research should focus on faster and more accurate models. This includes multimodal approaches (e.g., combining endoscopic video with intra-op US) to continuously correct models. Hybrid DL frameworks, e.g., physics-informed neural nets that learn organ elasticity, may offer both speed and realism. Transfer learning across organs could reduce the need for organ-specific training data. The ongoing P2ILF challenge exemplifies how community benchmarks can drive innovation in automated registration^[7]^. Open-source toolkits (such as the IBIS platform for neurosurgery) should incorporate deformation modeling modules to accelerate adoption. To accelerate reproducible progress, academic groups should prioritize publishing annotated multimodal datasets and lightweight, open deformation modules so others can reproduce and extend results.

### Clinical trials and validation studies

Prospective trials in target surgeries are essential. Initial feasibility studies should be followed by controlled studies comparing AR-guided vs standard navigation on endpoints like resection margins or operative time^[3]^. Multicenter consortia can help gather sufficient data. Objective metrics, e.g., image-based error tracking or post-op imaging overlays, will quantify benefit. Regulatory science initiatives (like the FDA’s Device Investigational Clinical Evaluation analysis for device trials) could tailor evaluation frameworks to AR’s unique blend of software and hardware. Clinicians should lead the selection of high-impact target indications, include human-factors endpoints (e.g., NASA-TLX, override frequency) in early trials, and organize multicenter protocols to produce generalizable evidence.

### Standardization efforts

Industry and academia should collaborate on common interface and data standards (e.g., Digital Imaging and Communications in Medicine for intra-op imaging, Image-Guided Surgery Toolkit/Public Library for Ultrasound for trackers). AR device manufacturers and surgical societies could form working groups to draft best-practice documents (similar to the pre-surgical checklists used in Ambulatory Surgical Centers). Video and image datasets should be made available (with de-identification) for algorithm benchmarking. Validation phantoms (e.g., deformable organ models with known ground truth) are also needed for calibration. Industry partners should commit to interoperable interfaces and publish validated calibration phantoms and reference implementations so hospitals and labs can benchmark components consistently.

### Regulatory and reimbursement pathways

Early engagement with regulators (FDA, European Medicines Agency) will clarify whether new AR algorithms need 510(k) clearance, De Novo, or Pre-market Approval. Demonstrating clinical utility, not just technical accuracy, will be key to insurance coverage. Health economic studies should be planned in tandem to show that improved navigation (fewer complications, shorter OR time) offsets system costs. International harmonization (e.g., via International Medical Device Regulators Forum) of software-as-medical-device guidelines will benefit multinational trials. Regulators should provide modular pre-submission guidance that recognizes component-level evidence (e.g., registration module versus full system) and clarify acceptable surrogate metrics for conditional pathways.

### Ethical and training frameworks

Guidelines for safe AR use must be developed. Human–AI collaboration should remain central, with AR designed to support rather than replace surgical judgment, ensuring transparency and easy override to prevent overreliance. This includes surgeon training certification (to avoid overreliance on AR), fail-safes (e.g., “AR off” triggers), and patient information protocols. Training should be staged and simulation-driven: combine instrumented physical phantoms, digital-twin scenarios (including deliberate failure modes), and scenario-based drills, with proficiency demonstration before supervised clinical use. Cybersecurity standards specific to wearable medical devices should be enforced. Research into the cognitive impact of AR (distraction, eyestrain) must continue, as must efforts to keep user interfaces simple and sterile-compatible.

### Technological enablers

Improvements in hardware, lighter headsets with better registration, faster GPUs, and integrated tracking cameras will ease AR adoption. 5G networking and edge computing could allow heavy computation (e.g., DL inference) off-device. AR (mixed reality) systems may incorporate novel feedback (haptics, gesture control) to enhance usability. Integration with robotic surgical systems is also a frontier: combining AR overlays with robot control (e.g., da Vinci) could automate certain tasks under surgeon supervision.

Coordinated action by academia, industry, clinicians, and regulators, along with shared datasets, standardized benchmarks, and staged training, is essential to turn deformation-aware AR into safe, effective clinical practice.

## Conclusion

AR-guided surgery is quickly advancing from idea to clinical application. Importantly, next-generation AR must account for organ deformation to ensure accuracy. A range of approaches from physics-based models to machine learning registrations is converging toward this goal. Early research already indicates that AR overlays can significantly enhance intraoperative localization and navigation. To fully achieve this potential, the field requires rigorous clinical validation and attention to translational challenges such as regulatory approval, standardization, and ethics. Without these efforts, AR risks remaining limited to research prototypes with minimal clinical trust, which could increase disparities in access and slow integration into surgical workflows. On the other hand, the proposed roadmap can foster multicenter collaboration, establish common benchmarks, and attract funding by aligning the priorities of academia, industry, and regulators. With ongoing and coordinated efforts, AR-driven deformation modeling could become a standard tool that enhances surgeons’ vision and enables safer, less invasive procedures globally.

## Data Availability

All data generated during this study are included in this published article.
